# The impact of mental health on health-related quality of life in patients with NF2-related Schwannomatosis

**DOI:** 10.1038/s41598-024-57401-7

**Published:** 2024-03-23

**Authors:** Anna Freier, Anna C. Lawson McLean, Denise Loeschner, Steffen K. Rosahl, Johannes Kruse

**Affiliations:** 1 Department of Psychosomatic Medicine and Psychotherapy, Phillips University Marburg, Marburg, Germany; 2 Department of Neurosurgery, Helios Klinikum Erfurt, Health and Medical University (HMU), Erfurt, Germany; 3 Department of Neurosurgery, University Hospital Jena, Jena, Germany; 4 Department of Psychosomatics and Psychotherapy, University of Giessen, Giessen, Germany

**Keywords:** Neurofibromatosis type 2, *NF2*-related schwannomatosis, Quality of life, Mental health, Depression, Rare disease, Neurological disorders, Neurological disorders, Anxiety, Depression, Quality of life

## Abstract

*NF2*-related schwannomatosis (*NF2*-SWN) is a rare genetic disorder and is associated with progressive morbidities. This study aimed to investigate the relationship between *NF2*-SWN disease severity, health-related Quality of Life (QoL), and mental health aspects of patients. Standardised questionnaires assessing mental health problems (symptoms of depression, anxiety, and somatic burden), psychological factors (resilience, loneliness, and personality functioning), and health-related QoL were administered to 97 patients with *NF2*-SWN. The results of these questionnaires were compared with physician-rated disease severity. Questionnaires were completed by 77 patients. Physician-rated disease severity scores were available for 55 patients. *NF2*-SWN patients showed a high prevalence of clinically relevant symptoms of depression (30%), anxiety (16%), and somatic burden (32%). Almost all variables showed moderate to high correlations with *NF2*-SWN-related QoL. *NF2*-SWN-related QoL was associated with physician-reported disease severity (*r* = 0.614). In the stepwise hierarchical linear regression analysis, a significant model with four predictors (disease severity type, depression symptoms, personality functioning, and gender) explained 64% of the variance in *NF2*-SWN-related QoL. Our results showed a strong association between *NF2*-SWN-related QoL and depression symptoms. Moreover, personality functioning is an important influencing factor, representing a modifiable construct that can be targeted by prevention programs or psychotherapy.

## Introduction

Rare genetic diseases traditionally receive little attention from health authorities, clinicians, and researchers, although their medical handling and treatment are complex, and their influence on an individual’s life is often severe. Studies have shown that patients with rare diseases have a poor health-related quality of life (QoL)^1^ and a high proportion of mental health problems^2^. Although the relationship between disease severity and QoL has been well researched, there is limited evidence on the impact of mental health problems on QoL in rare diseases.

*NF2*-related schwannomatosis (*NF2-*SWN) is one such rare autosomal dominant disorder with complete penetrance and is characterised by the development of multiple benign tumours of the central and peripheral nervous system^3^. Patients with *NF2*-SWN develop multiple benign cerebral and spinal tumours that may lead to symptoms like hearing loss, balance disturbances, impaired vision, and facial weakness. Disease progression in *NF2*-SWN is monitored in terms of neurological deficits, neuroimaging, and speech and pure-tone audiometry. The patients with *NF2*-SWN undergo a combination of available treatment options including surgical extirpation, radiotherapy, and off-label immunotherapy to mitigate symptom burden and improve health-related QoL.

Over the last decade, health-related QoL has gained importance in healthcare and research. Health-related QoL is defined as a multidimensional construct that consists of at least three broad domains such as physical, psychological, and social functioning that are affected by one’s disease and/or treatment^4^. In the case of *NF2*-SWN, only a few studies have investigated QoL^5–9^. Overall, the general QoL of patients with *NF2*-SWN is lower than that of the general population and similar to that of patients with cancer^5,6^. Hornigold et al. (2012) developed a *NF2*-SWN-specific QoL questionnaire that showed similar results of considerable burden^6–9^. Furthermore, health-related QoL was highly correlated with physician-rated disease severity (r = 0.512)^7^ and patient-reported disease severity (r = 0.52—r = 0.62)^8^. However, in this relationship the impact of psychological and social aspects is not investigated.

To date, few studies have shown elevated psychological distress and its negative impact on social relationships in patients with *NF2*-SWN^5,10,11^. Hamoy-Jimenez et al. (2020) showed a relationship between the QoL (SF-36) and pain, anxiety, and depression symptoms. Moreover, increased symptoms of depression and anxiety and higher levels of perceived stress have been associated with a higher frequency of self-reported medical visits in the past year^12^. To date, there are no available data on the psychological and social aspects related to health-related QoL in German *NF2*-SWN patients. Moreover, this is the first study to investigate this relationship with regard to disease severity in *NF2*-SWN patients.

This study aimed to evaluate the mental health problems in *NF2*-SWN patients and investigate their relationships on health-related QoL with respect to disease severity. Therefore, we chose various mental health indicators, namely, depression, anxiety, and somatic symptoms. Loneliness, resilience, and personality functioning were measured as potential influencing factors.

## Materials and methods

The study was conducted as an extension of the Erfurt Neurofibromatosis Database Research (NF registry), which contains disease data of patients with diagnosed *NF2*-SWN. This database is an online registry on the Castor EDC platform, which is in accordance with European data protection laws. The registry was approved by the regional ethics review board and collects pseudonymized patient data. It is regularly updated at each in-patient or out-patient visit. The investigation was approved by the local Ethics Committee of Erfurt (ref:2278/2020/6). All patients provided written informed consent. STROBE guidelines for cross sectional studies were used^13^. The study was conducted in accordance with the Declaration of Helsinki.

An open web-based survey was conducted from December 2021 to January 2022, using the SoSci Survey online tool. *N* = 97 patients at the Erfurt Neurofibromatosis Center were invited via email to participate voluntarily in an online survey. The survey included questions regarding age, gender, and date of birth. The date of birth from the survey data was required to match the data from the NF registry. Inclusion criteria were the diagnosis of “*NF2*-related Schwannomatosis” and a minimum age of 16.

### Disease severity

The clinician-reported disease severity score consists 7 symptoms with major impact of patients life: (1) loss of hearing on both ears; (2) severe visual impairment on both eyes; (3) bilateral facial paralysis, on at least on side ≥ H&B°3; (4) depression/anxiety disorder; (5) severe chronic pain / substance abuse due to pain; 6) immobility; and (7) malignancies^9^. In the absence of all points the severity rated as mild, 1–3 symptoms are indicated moderate severity and 4–7 symptoms are reported in the case of severe *NF2*-SWN. The disease severity score ranging from 1 (= mild) to 3 (= severe). The participants’ disease severity was rated based on the NF registry data. The disease severity score was derived from the latest clinical data available in the NF registry. This score has been validated (publication currently under review), and a version of it is detailed in a recent publication^9^.

### Health-related quality of life

Hornigold et al. (2012) developed the Neurofibromatosis 2 Impact on Quality of Life (NFTI-QoL) questionnaire to evaluate health-related QoL in *NF2*-SWN patients. This eight-item questionnaire assesses different disease-specific domains, such as balance, hearing, facial weakness, vision, mobility/walking, role/outlook in life, pain, and anxiety/depression. Each item consists of a four-point scale ranging from 0 to 3, with 3 being the most impaired. The maximum total score is 24; the higher the NFTI-QoL score, the worse the outcome. The German version (NFTI-QoL-D) demonstrated metric properties comparable to those of the English-speaking version^9^.

### Mental health problems

#### Depression symptoms

The nine-item Patient Health Questionnaire (PHQ-9) is a self-administered survey developed for depression^14^. Each of the items of the PHQ-9 is quantified on a four-point Likert scale (0 = “not at all”—3 = “nearly every day”). The total score ranged from 0 to 27, with 0 indicating no symptoms of depression and 27 indicating that all symptoms occur almost daily.

The PHQ-9 has excellent test–retest reliability and excellent criterion and construct validity^14^. The internal consistency has been validated (Cronbach’s α = 0.89). Moreover, threshold scores exist to identify different depression severity levels, including minimal (0–4), mild (5–9), moderate (10–14), moderately severe (15–19), and severe (≥ 20) depression. A score of ≥ 10 has been shown to have an 88% sensitivity and 88% specificity for major depression in the general medical population^14^.

#### Anxiety symptoms

The Generalised Anxiety Disorder Questionnaire (GAD-7) is a seven-item self-reported scale developed to evaluate the symptoms of generalised anxiety disorder^15^. The items are rated on a four-point Likert scale (0 = “not at all”—3 = “nearly every day”). The GAD-7 items describe some of the most important diagnostic criteria for generalised anxiety disorder (i.e. feeling nervous, anxious, or on edge, and worrying too much). The total score ranged from 0 to 21, with higher scores indicating more severe generalised anxiety disorder symptoms. Studies have proposed that the GAD-7 is a valid screening tool for generalised anxiety disorder and for assessing its severity in clinical practice and research^15,16^. The GAD-7 also defines threshold scores for different severity levels: minimal (0–4), mild (5–9), moderate (10–14) and severe (15–21) anxiety symptoms. A GAD-7 total score of ≥ 10 represents a moderate to severe level of generalised anxiety and is indicative of a suspected diagnosis of generalised anxiety disorder. The GAD-7 showed good sensitivity (89%) and specificity (82%) for detecting generalised anxiety disorder in primary care patients, and its internal consistency was excellent (Cronbach’s α = 0.92).

#### Somatic symptoms

The burden of somatic symptoms was measured using the Somatic Symptoms Scale (SSS-8), a self-report questionnaire. The SSS-8 was developed as an abbreviated eight-item version of the PHQ-15 to assess the presence and severity of common somatic symptoms^17^. The SSS-8 assesses the severity of the following somatic symptoms experienced by the respondent during the past seven days: (1) stomach or bowel problems; (2) back pain; (3) pain in the arms, legs, or joints; (4) headaches; (5) chest pain or shortness of breath; 6) dizziness; (7) feeling tired or having low energy; and (8) having trouble sleeping. Each item is quantified on a five-point Likert scale (0 = “not at all”—4 = “very much”). The total score ranges from 0 to 32, with higher scores indicating more somatic symptoms. Severity threshold scores define five different burden levels of somatic symptoms: no to minimal (0–3), low (4–7), medium (8–11), high (12–15), and very high (16–32) somatic symptoms. High somatic symptom burden was the case between the 95th and 98th percentiles. The German version of the SSS-8 has been validated in the general German population^18^.

### Psychological factors

#### Resilience

The 13-item Resilience Scale (RS-13) quantifies resilience on a seven-point scale^19^. Individuals rate different statements (from 1 = “I do not agree” to 7 = “I agree completely”). RS-13 is a short German version of RS-25^20^. The scores range from 13 to 91, with higher scores indicating higher resilience. Based on the reference groups, individuals with < 72 points on the resilience scale (RS-13) are defined as individuals with low resilience. Highly resilient individuals have scores ≥ 72^19^.

#### Loneliness

The Loneliness Scale^21^ consists of three items, introduced by “How often do you feel…”: (1) “…that you lack companionship?”; (2) “…left out?”; (3) “…isolated from others?”. Items are rated on a five-point Likert scale (0 = “never” to 4 = “very often”). Responses are added up to obtain a total score of 0–12, with higher scores indicating a higher level of loneliness. The German version of the Three-Item Loneliness Scale was validated in a representative sample, and norm values are reported^22^.

#### Personality functioning

Personality functioning describes a person’s abilities in four domains related to cognition/perception, regulation, communication, and attachment^23^. Individuals with impaired personality functioning tend to suffer from severe disturbances of the self and their interpersonal relationships and have an increased risk of developing mental disorders such as depression and anxiety^24^. Personality functioning was measured with the short version of the Operationalized Psychodynamic Diagnosis-Structure Questionnaire (OPD-SQS)^25^. It is a self-report questionnaire for screening personality dysfunction^26^. The OPD-SQS consists of a 0–4 Likert scale (0 = “fully disagree” to 4 = “fully agree”). It measures three highly correlated subscales: self-perception, interpersonal contact, and relationship model. The total score ranges from 0 to 48. Lower scores on the OPD-SQS indicate better personality functioning, whereas higher scores on the OPD-SQS indicate impairments in personality functioning.

### Statistical analyses

Data analyses were performed using IBM SPSS (version 20). Since single values were missing from the questionnaires, the mean substitution method was applied. Isolated single missing values in the questionnaires were replaced using the rounded individual means of the respective questionnaires. This was done once for each case for the PHQ-9, SSS-8, and OPD-SQS. Patients with more missing items in the same questionnaire were excluded from the questionnaire analysis. This was done once for each case for the GAD-7 and SSS-8. Missing values in the NFTI-QoL-D were replaced with rounded item means. Descriptive statistics were calculated for age, gender, *NF2*-SWN-related QoL, depression, anxiety, somatic symptoms, resilience, loneliness, and personality function.

First, Pearson’s correlations between different variables were calculated to examine the associations. Second, a stepwise hierarchical linear regression analysis was performed. This included determining *NF2*-SWN-related QoL as a dependent variable. Three groups of variables served as predictors and were entered stepwise into the equation. The first predictors were disease severity score and gender. At step 2, values of resilience, loneliness, and personality functioning were entered stepwise as potential psychological predictors. The third group of variables included scores for depression, anxiety, and somatic symptoms as indicators of mental health problems, which are also entered stepwise. Adjusted R squares (R^2^) and standardised regression coefficient (β) were reported. The results were considered significant at *p* < 0.05.

## Results

### Participants

A total of 77 patients completed the online survey. The mean age was 37.6 years (range 16–68 years), and 63.6% (N = 49) of the participants were female. Current disease severity data of 55 patients were available. Physician-rated disease severity scores identified 24 patients with mild disease, 27 with moderate disease, and 4 with severe disease. In the comparison between *NF2*-SWN patients with and without a disease severity score rating, a significant difference was found only in age [*t*(75) = 3.433, *p* = 0.001]. *NF2*-SWN patients without a rating of disease severity score were approximately 10 years younger, on average (*M* = 30.50 vs. *M* = 40.42). All other parameters, including gender, psychological aspects, and mental health problems, did not show significant differences.

### Outcome *NF2*-SWN-related QoL

The outcomes of the *NF2*-SWN-related QoL are shown in Table 1. 58 *NF2*-SWN patients (75%) reported that “the role of the disease on outlook on life” had a moderate or large negative effect. A second major impairment in the QoL, as reported by two-thirds of *NF2*-SWN patients (66%, n = 51), was “hearing problems or hearing loss”, causing difficulties or stoppage of their usual activities. A significant association between *NF2*-SWN-related QoL and the disease severity score was observed in ANOVA [*F*(52,2) = 15.747, *p* < 0.001]. The mean NFTI-QoL-D in the mild disease severity type was *M* = 7.21 [standard deviation (*S*D) = 3.38] which was significantly lower than the mean NFTI-QoL-D in the moderate disease severity type [*M* = 12.19, *S*D = 4.51; *t*(49) = -4.418, *p* < 0.001]. The severe disease severity type showed the worst *NF2*-SWN-related QoL *M* = 16.50 (SD = 0.867) in the NFTI-QoL-D, which was significantly different from that of the moderate disease severity type [*t*_Welch_(28.996) = − 4.720, *p* < 0.001].

**Table 1:** Results of health-related quality of life in 77 *NF2*-SWN patients.

Symptoms/Signs	Not present n (*%*)	Yes, but no difficulties n (*%*)	Yes, and cause some difficulties n (*%*)	Yes, it stops my usual activities n (*%*)
Q1. Hearing	4 (5%)	22 (29%)	36 (46%)	15 (20%)
Q2. Dizziness and balance	12 (16%)	28 (35%)	26 (34%)	11 (15%)
Q3. Facial palsy	30 (39%)	28 (36%)	14 (18%)	5 (7%)
Q4. Sight problems	25 (32%)	33 (43%)	15 (20%)	4 (5%)
Q5. Mobility and walking	No problems n (*%*)	Some difficulty, can manage on own n (*%*)	Unable to walk without help n (*%*)	Unable to walk at all n (*%*)
	33 (43%)	34 (44%)	10 (13%)	0 (0%)
Q6. Role and outlook on life	Positive or no effect n (*%*)	Small negative effect n (*%*)	Moderate negative effect n (*%*)	Large negative effect n (*%*)
	10 (13%)	22 (29%)	33 (42%)	12 (16%)
Q7. Pain	None n (*%*)	Mild n (*%*)	Moderate n (*%*)	Severe n (*%*)
	21 (27%)	19 (25%)	28 (36%)	9 (12%)
Q8. Anxiety and depression	None n (*%*)	Mild n (*%*)	Moderate n (*%*)	Extreme n (*%*)
	38 (49%)	23 (30%)	11 (14%)	5 (7%)

Data are represented as frequency (percentage), n (%).

### Outcome mental health problems

The critical cutoff value in the mental health questionnaire was determined (Table 2). *NF2*-SWN patients with a moderate and severe disease types exceeded the critical value in the mental health questionnaires. Overall, for depression, n = 17 (31%); for anxiety, n = 9 (16%); and for somatic symptoms burden, 18 (33%) *NF2*-SWN patients exceeded the critical cutoff value.

**Table 2:** Proportion of NF2-SWN patients above PHQ-9, GAD-7, and SSS-8 Cut-offs.

Questionnaires	Mild (n = 24)	Moderate (n = 27)	Severe (n = 4)	Total
Depression symptoms	5 (17.4%)	9 (33.3%)	3 (75.0%)	17 (30.9%)
Anxiety symptoms	2 (4.3%)	6 (23.1%)	1 (25.0%)	9 (16.4%)
Somatic symptoms burden	5 (17.4%)	11 (40.7%)	2 (50.0%)	18 (32.7%)

Data are represented as frequency (percentage), n (%).

*PHQ-9* depression symptoms, *GAD-7* anxiety symptoms, *SSS-8* somatic symptoms burden.

The results of mental health problems in relation to disease severity were heterogeneous. A continuous increase in depression symptoms was observed. Thus, the higher the severity of the disease, the more depression symptoms were measured in *NF2*-SWN patients. In the case of anxiety and somatic symptoms burden, an increase was only notable between the mild and moderate disease severity types. Analysis of variance (ANOVA) showed no significant differences in depression symptoms [*F*(52,2) = 0.959, *p* = 0.390], anxiety [*F*(51,2) = 0.164, *p* = 0.849], or somatic symptoms burden [*F*(51,2) = 1.764, *p* = 0.182].

The means and standard deviations of the all utilized questionnaires in relation to disease severity are shown in Table 3.

**Table 3:** All questionnaires presented for each disease severity.

	Mild (n = 24) *M* (SD)	Moderate (n = 27) *M* (SD)	Severe (n = 4) *M* (SD)
NF2-SWN-related quality of life	7.21 (3.38)	12.19 (4.51)	16.50 (0.58)
Depression symptoms	6.46 (5.70)	8.11 (4.89)	9.50 (3.87)
Anxiety symptoms	5.46 (4.26)	6.23 (5.55)	6.25 (4.50)
Somatic symptoms burden	7.67 (4.91)	10.85 (7.14)	10.75 (5.91)
Resilience	65.38 (14.98)	66.22 (12.64)	77.75 (8.06)
Loneliness	5.71 (3.99)	7.07 (3.23)	7.50 (4.04)
Personality functioning	19.08 (9.67)	18.93 (9.98)	21.75 (13.87)

*n* numbers, *M* means, *SD* standard deviations.

### Correlation analysis

Table 4 presents Pearson’s correlation coefficients between all variables. *NF2*-SWN-related QoL correlated with almost all variables. There were strong correlations between *NF2*-SWN-related QoL and disease severity scores (r = 0.614), depression symptoms (r = 0.583), as well as somatic symptoms burden (r = 0.579). Notably, there were no further significant associations with disease severity. The strongest correlation was observed between depression and somatic symptoms burden (r = 0.802).

**Table 4:** Descriptive statistics and pearson correlations of *NF2*-SWN-related quality of life, disease severity scores and all questionnaires.

	n	M (SD)	2	3	4	5	6	7	8
1. NF2-SWN-related quality of life	77	9.99 (4.55)	0.614***	0.583***	0.489***	0.579***	− 0.154	0.392***	0.421***
2. Disease severity score	55	1.64 (0.62)		0.188	0.073	0.230	0.166	0.190	0.039
3. Depression symptoms	77	7.58 (5.56)			0.729***	0.802***	− 0.496***	0.524***	0.532***
4. Anxiety symptoms	76	5.67 (4.41)				0.551***	− 0.468***	0.503***	0.632***
5. Somatic symptoms burden	76	9.75 (6.27)					− 0.368**	0.431***	0.440***
6. Resilience	77	66.32 (13.61)						− 0.524***	− 0.597***
7. Loneliness	77	6.17 (3.49)							0.649***
8. Personality functioning	77	19.25 (9.72)							

Significant values are in bold.

*p < .05, **p < .01, ***p < .001.

*n* numbers, *M* means, *SD* standard deviations.

### Regression analysis of health-related QoL, psychological factors, and mental health

Stepwise hierarchical multiple regression analysis was conducted to examine whether psychological factors (resilience, loneliness, and personality functioning; Step 2) and mental health indicators (depression, anxiety, and somatic symptoms burden; Step 3) could predict health-related QoL in *NF2*-SWN patients (Table 5). We accounted for the influence of gender and disease severity. In the final model, 64% of the variance in *NF2*-SWN-related QoL was explained by four predictors (disease severity score, depression symptoms, personality functioning, and gender).

**Table 5:** Models of stepwise hierarchical multiple regression analysis for gender, disease severity type, personality functioning, and depression symptoms as predictors of *NF2*-SWN-related quality of life.

Variable	*B*	*SE B*	*β*	*R*	*R*^*2*^
Step 1				0.67	0.45
Gender	− 2.48	4.04	− 0.25*		
Disease severity type	4.59	0.84	0.58***		
Step 2				0.74	0.55
Gender	− 1.36	1.03	− 0.14		
Disease severity type	4.58	0.77	0.58***		
Personality functioning	0.16	0.05	0.33**		
Step 3				0.80	0.64
Gender	− 0.84	0.94	− 0.09		
Disease severity type	4.1	0.71	0.52***		
Personality functioning	0.06	0.06	0.11		
Depression symptoms	0.37	0.11	0.39**		

N = 55; *B* = regression coefficient; *SE* (*B*) = standard error of regression coefficient; *β* = standardised regression coefficient.

*p < .05, **p < .01, ***p < .001.

The first significant model [*R*^2^ = 0.45, *F*(2, 50) = 20.60, *p* < 0.001] showed disease severity [*β* = 0.58, *t*(50) = 5.50, *p* < 0.001] and gender [*β* = -0.25, *t*(50) = − 2.36, *p* = 0.022] as significant predictors of *NF2*-SWN-related QoL. In the second step, personality functioning was introduced as a significant predictor (*β* = 0.33, *t*(49) = 3.20, *p* = 0.002), while the effect of gender became nonsignificant in this step. Including depression symptoms as a significant mental health predictor [*β* = 0.39, *t*(48) = 3.49, *p* = 0.001] led to a third significant model [*R*^2^ = 0.64, *F*(4, 48) = 21.16, *p* < 0.001]. In this step, personality functioning also lost statistical significance.

### Post hoc analyses

**Figure 1:**
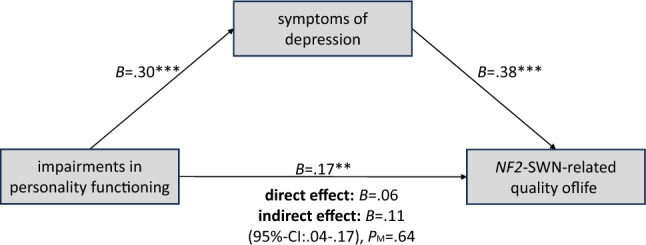
Mediating model of symptoms of depression in the relationship between impairments of personality functioning and *NF2*-SWN-related quality of life. Unstandardized coefficients are reported. *p < 0.05; **p < 0.01; ***p < 0.001; CI = confidence interval (percentile-bootstrap, 5000 samples); P_M_ = proportion of the mediating effect as proportion of the total effect.

Post hoc mediation and moderation analyses were conducted to investigate the associations of gender and personality functioning to *NF2*-SWN-related QoL. For gender and *NF2*-SWN-related QoL, no significant moderator or mediator analyses were found, neither for personality functioning nor for symptoms of depression. For personality functioning and *NF2*-SWN-related QoL, a significant mediation analysis was conducted (Fig. 1). No significant moderation effect was found in this case.

## Discussion

The major finding of the present study is the strong association between health-related QoL and depression symptoms in *NF2*-SWN patients. Moreover, by adding disease severity score, gender, and personality functioning as further important influencing factors, the model could explain 64% of the variance in *NF2*-SWN-related QoL. This is the first study that shows a strong association between disease severity, *NF2*-SWN-related QoL, and psychological components.

This finding is consistent with previous studies suggesting a high impact of mental health problems in *NF2*-SWN patients^6,12^. The present investigation replicated the results of the high proportion of depression and anxiety symptoms in patients with *NF2*-SWN, as reported in the study by Wang et al. (2012), and expanded the findings to include somatic symptoms burden. Moreover, Hamoy-Jimenez et al. (2020), reported a high proportion of anxiety/depression (68%); however, this refers to any type of anxiety/depression symptom. In the present study, one-third of *NF2*-SWN patients reported critical values for depression and somatic symptoms burden. Anxiety symptoms are present in approximately one out of every six *NF2*-SWN patients. In our cohort of *NF2*-SWN patients, the scores for depression (M = 7.58) and anxiety (M = 5.67) were significantly higher than the population norms (µ = 3.56 and µ = 2.95, respectively) (35, 36) but similar to other rare chronic diseases. Moreover, concerning critical values, the difference is even more pronounced. In the German norm population, the criterion for a depressive syndrome is fulfilled by 9.2%, while in the *NF2*-SWN cohort, this is the case in 31%. For anxiety symptoms, critical values were observed in approximately 5% of the German norm population, while 16% of patients with *NF2*-SWN surpassed the threshold. Clinicians should be aware of the high proportion of mental health problems and consider the need for psychosocial support. For example, a systematic monitoring survey of mental health problem symptoms can be introduced into medical investigations. For critical values, qualified professionals should be consulted. They can diagnose the relevant symptoms and, if necessary, initiate drug and/or psychotherapeutic treatment. This treatment should be investigated in the future.

Furthermore, the results of the present study are in line with the finding of a relationship between QoL and mental health problems^6^ and specify this for health-related QoL in *NF2*-SWN patients. Interestingly, besides the disease severity, depression symptoms showed the strongest association with quality of life in *NF2*-SWN. This could be due to the differentiation of the burden when the queried symptoms are present, i.e., whether they are rated as “no difficulties” or “cause some difficulties”. Therefore, the perception of difficulties may be more pronounced in *NF2*-SWN patients with depression symptoms. Moreover, the ability to cope with these limitations may be reduced in *NF2*-SWN patients with depression symptoms. Therefore, the treatment of depression symptoms could be an important approach to increasing *NF2*-SWN-related QoL.

The impact of personality functioning on *NF2*-SWN-related QoL is also noteworthy. In the model building, these psychological factors show at first a positive association with *NF2*-SWN-related QoL, which diminishes when considering depression symptoms. In the post hoc analyses, a mediation effect of depression symptoms in the relationship between personality functioning and *NF2*-SWN-related QoL was identified. This implies that personality functioning influences depression symptoms, and depression symptoms, in turn, influence *NF2*-SWN-related QoL. Only a small direct association between personality functioning and *NF2*-SWN-related QoL remained. Previous studies have shown that personality functioning is associated with depression^24^. However, personality functioning can be modified through psychotherapy^27^ and these changes in personality functioning may contribute to the improvement of depressive symptoms^28^. Hence, personality functioning should be taken into consideration in treatment or prevention programs.

Another finding was the increased prevalence of somatic symptoms burden in *NF2*-SWN patients compared to that in the general population^18^. It should be noted that many somatic symptoms that are queried in the SSS-8 can also be caused by various benign tumours. However, no significant correlation between disease severity and somatic symptoms burden was found in this study (r = 0.230), whereas the association between depression symptoms and somatic symptoms burden was very high (r = 0.802). This is consistent with previous studies, which have already found a comparably high correlation between somatic and depression symptoms^18^. This finding supports the assumption that somatic problems are more likely related to increased depression symptoms. This result is also consistent with the findings of Wang et al. (2012), who observed an association between increased depression symptoms and a higher frequency of self-reported medical visits. However, in our study one in three *NF2*-SWN patients described having a clinically relevant burden of somatic symptoms. Among *NF2*-SWN patients with objectively mild disease severity (i.e. without major *NF2*-SWN symptoms), 17% reported a subjectively high burden of somatic symptoms. This might correspond to the fact that just living with a rare disease leads to a perceived burden^29^. Therefore, clinicians should be aware that increased somatic symptoms burden can indicate mental health problems.

This study has some potential limitations. The number of *NF2*-SWN patients with data for their disease severity scores was small, but comparable to other *NF2*-SWN studies. This resulted in low statistical power, especially in inter-group statistics. Therefore, differences in depression, anxiety, and somatic symptoms burden between different disease severities may have been overlooked. Further studies with larger sample sizes are required to confirm this hypothesis. With larger samples, it would also be possible to explore complex relationships using a Structural equation modeling. In this study, mental health problems were assessed only by self-reported questionnaire data. Unfortunately, there was no professional assessment by a psychiatrist, which is recommended for future studies, to determine whether disease-relevant symptoms are present. Further validation of the effectiveness of this classification system is required through additional independent researchers. Another potential confounding factor in the study is the duration of the disease, defined as the time between diagnosis and assessment. Furthermore, it is correlated with the age of the patients, as most individuals are diagnosed between the ages of 20 and 30. Both of these factors should be considered in future studies.

This study was based on sound clinical data and a validated disease severity scale. Moreover, the study used standardised and well-established measurement tools for mental health problems and potential influencing factors.

In summary, this investigation suggests the importance of conducting mental health screenings for individuals with *NF2*-SWN. Furthermore, these findings have potential value for patients with other rare diseases and can serve as inspiration for future research in the field.

## Data Availability

Individual deidentified participant data (including data dictionaries) will be shared on request, beginning 9 months and ending 36 months following article publication. Related documents will be available (study protocol, statistical analysis plan) will also be available. Data will be shared with researchers for scientific analysis only via a dedicated closed data room. For contact details, please refer to the following link: https://www.helios-gesundheit.de/kliniken/erfurt/unser-angebot/unsere-fachbereiche/neurochirurgie/data-sharing/.
